# Myocardial, Valvular and Vascular Abnormalities in Repaired Tetralogy of Fallot

**DOI:** 10.3390/life14070843

**Published:** 2024-07-03

**Authors:** Attila Nemes

**Affiliations:** Department of Medicine, Albert Szent-Györgyi Medical School, University of Szeged, Semmelweis Street 8, P.O. Box 427, H-6725 Szeged, Hungary; nemes.attila@med.u-szeged.hu; Tel.: +36-62-545220; Fax: +36-62-544568

**Keywords:** myocardial, mechanics, vascular, valvular, repair, tetralogy of Fallot

## Abstract

Tetralogy of Fallot is the most common heart defect associated with cyanosis characterized by the co-occurrence of pulmonary stenosis, right ventricular hypertrophy, and ventricular septal defect with over-riding of the aorta. The present review purposed to summarize myocardial, valvular and vascular abnormalities, which were described in a series of patients following repair of tetralogy of Fallot. It was also aimed to describe potential differences in these parameter using different surgical strategies.

## 1. Introduction

Tetralogy of Fallot (TOF) is the most common congential heart defect (CHD) associated with cyanosis characterized by the co-occurrence of pulmonary stenosis, right ventricular (RV) hypertrophy, and ventricular septal defect with over-riding of the aorta [1]. Its frequency considering all heart defects is 3.5%, while considering all live births, its prevalence is 1/3600. Its cause is unknown, and most cases occur sporadically, but family accumulation can also be observed. Its association with certain genetic abnormalities, however, has been described including 22q11-micro deletion, the mutation underlying DiGeorge syndrome, which can be detected in almost a quarter of patients [2].

In recent decades, two surgical strategies have been used. Previously, the size of the patient and the techniques available at that time only allowed for a palliative surgical solution. In such cases, systemo-pulmonary shunts were established (Blalock–Taussig, Potts, Waterston–Cooley) in order to ensure the blood supply of the pulmonary circulation and thus the survival of the patient. After reaching the body size required, the complete reconstruction could be performed in a second session with a closure of the ventricular septal defect and restoration of the RV outflow tract. However, in such cases, there is a risk that it will already progress and cause irreversible morphological and functional transformation of the right side of the heart by the time the second surgery is performed. Owing to developing surgical techniques and perioperative care, early full reconstruction can now be performed even in patients with very low body weight. In the long term, early complete reconstruction carries the risk that the child will “outgrow” the pulmonary homograft (pulmonary valve replacement, PVR) early, and thus reoperation may be necessary several times during his life [1,3,4].

## 2. Cardiovascular Imaging and Criteria

In addition to what has been written above, it is a well-known fact that cardiovascular imaging has also undergone a significant development. The importance of this in the case of repaired TOF (rTOF) is that complicated anatomical conditions can now be diagnosed with high precision in a non- or semi-invasive way. Considering that TOF is a rare disease, this is of fundamental importance. Not only has echocardiography been developed significantly, and new speckle-tracking (STE) and/or three-dimensional (3D) techniques been spread all around the world, but cardiac magnetic resonance imaging (cMRI) and computer tomography have become part of the daily routine with significant importance, usability, applicability and reproducibility.

3DSTE is a relatively new echocardiographic technique which enables the 3D evaluation of complicated anatomical conditions using a virtual 3D echocardiographic cast and assessment of valvular annuli [5,6,7,8]. In the present summary, findings from our ‘Motion Analysis of the heart and Great vessels bY three-dimensionAl speckle-tRacking echocardiography in Pathological cases’ [MAGYAR-Path] Study are also presented, aiming to examine 3DSTE-derived parameters in certain disorders including rTOF since 2011 [9,10,11,12,13,14]. Clinical data of these patients originate from the Registry of C(S)ONGenital caRdiAc Disease patients at the University of Szeged (CSONGRAD Registry), which summarizes data of almost 4000 CHD patients who have been treated and managed since 1961 at the University of Szeged, Hungary [15].

The purpose of the present review was to summarize the results of examinations considering myocardial, valvular and vascular abnormalities in patients with rTOF emphasizing differences following different surgical strategies including findings from our own MAGYAR-Path Study. From vascular parameters, only aortic and pulmonary artery-related abnormalities were detailed.

TOF is frequently associated with disorders like atrial fibrillation and heart failure, non-invasive/invasive treatment of these conditions was not managed separately in the text due to limited information. Different types of surgical/interventional methods within the treatment groups were not managed separately either. Rare TOF-associated other abnormalities and case reports were not listed in this paper either.

## 3. The Left Heart and the Aorta

### 3.1. Left Ventricle

#### 3.1.1. Under Healthy Circumstances

**Figure 1:**
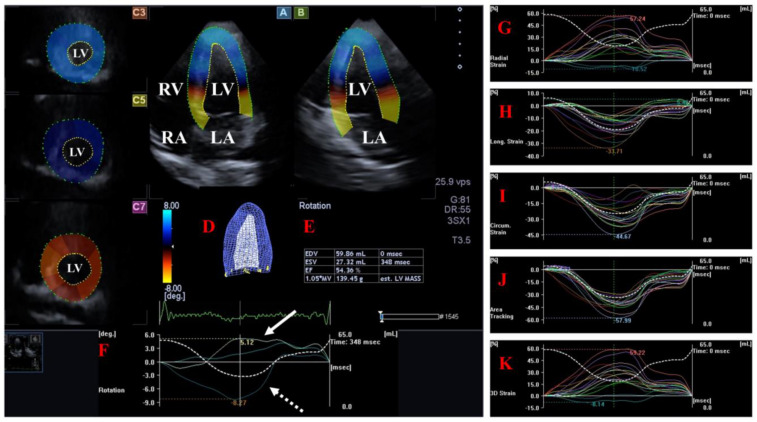
Examination of the left ventricle (LV) by three-dimensional (3D) speckle-tracking echocardiography. Following echocardiographic data acquisitions, the following typical views are created: (**A**) Apical 4-chamber and (**B**) two-chamber longitudinal views and (**C3**,**C5**,**C7**) cross-sectional views at apical, midventricular and basal levels, respectively. LV can be easily detected alongside other heart cavities including the left atrium (LA) and the right atrium (RA) and ventricle (RV). A number of other details were also presented including (**D**) 3D cast and (**E**) end-diastolic (EDV) and end-systolic (ESV) volumes of the LV together with ejection fraction (EF) and mass of the LV and (**F**) curves representing changes in volumes and strains of LV over time. (**F**) Apical [white arrow] and basal [white dashed arrow] LV rotations and (**G**) radial, (**H**) longitudinal, (**I**) circumferential, (**J**) area and (**K**) 3D strains are also demonstrated.

The LV is the central engine of systemic circulation. The two papillary muscles of the LV are required for the proper function of the mitral valve (MV) separating the LV and the left atrium (LA) and allowing blood flow from the LA into the LV during diastole. The blood leaves the LV via the aortic valve (AV), which prevents the backflow of blood from the aorta to the LV during diastole under healthy circumstances. The fibers in the subepicardium are left-handed, the mid-layer fibers run in the circumferential direction, while the fibers in the subendocardium are right-handed [16]. The LV moves in a 3D pattern including radial, circumferential and longitudinal deformation. This sort of movement can be characterized by several quantitative parameters named echocardiographic (unidirectional strains represented by its 3D motion: radial (LV-RS), longitudinal (LV-LS) and circumferential (LV-CS). While area (LV-AS) strain combines LS and CS, 3D (LV-3DS) strain combines all unidirectional strains [5,6,7,8,17,18,19,20]. In addition to the above, LV has a movement similar to wringing a towel called LV twist. In this case, the LV base rotates in a clockwise direction, while the LV apex rotates in a counterclockwise direction in systole [17,18,19] [Figure 1].

#### 3.1.2. In Repaired Tetralogy of Fallot

##### LV Structure, Volumes and Function

LV (and RV) shape and function show abnormalities in rTOF [21]. Moderate or severe LV (or RV) systolic dysfunction shows an independent association with deteriorated clinical status following repair of TOF [22]. In rTOF patients, LV ejection fraction (EF) was negatively related to the RV end-systolic volume normalized to body surface area [23]. In NYHA class 1 rTOF patients, a frequently seen impaired systolic and diastolic LV function, LV adverse remodeling with LV atrophy, a decreased mass/volume ratio, and extracellular matrix expansion suggest cardiomyopathic changes. The best predictor for LV systolic dysfunction was the RV mass/volume ratio [24]. Currently, follow-up of patients is based on echocardiography and cMRI, although in a retrospective study, it was found that biventricular shape modes discriminated differences between rTOF patients who did and did not require subsequent PVR better than standard cMRI-based indices in current use [25]. There is an impaired contractile reserve for LV (and RV) in rTOF represented by exercise stress cMRI [26].

##### LV Strains

rTOF is associated with impaired LV deformation represented by LV strains [9,27,28,29,30]. In rTOF patients with normal LV-EF reduced two-dimensional (2D) STE-derived LV strain, especially segmental and global LV-CS and LV-LS could be detected [29,31,32]. Results from the MAGYAR-Path Study confirmed that TOF patients late after early total reconstruction with preserved LV strains showed supernormal mean segmental LV-RS and LV-3DS. In TOF patients late after early palliation/late correction all LV strains were decreased, and mostly septal segmental strains showed reductions. These findings could be explained by the presence of compensatory effects and can be traced back to the nature of TOF, the presence of the ventricular septal defect and the interventricular septal patch [9]. It has been partly confirmed in a 3DSTE-based study, in which reduced global LV-AS was found in rTOF [33]. Moreover, LV strains and aortic stiffness correlated as well [9]. In rTOF patients, global LV-RS, LV-LS and LV-CS were reduced, and global LV-CS reduction was more pronounced in patients with increased RV-ESV with preserved global LV-RS and LV-LS [31]. Shortly after surgical repair of TOF in children, despite normal LVEF, patients exhibit impaired LV strain and strain rate together with RV parameters which can have prognostic implications [34]. In rTOF patients, LV septal strain is reduced suggesting adverse effects of RV dysfunction on LV function [35]. Moreover, detailed analysis confirmed segmentally/regionality of strain abnormalities [36]. LV and RV function and strain were found to be associated and interact closely as well [29,37,38], while postoperative global LV-LS is more reduced compared to preoperative values in children with rTOF [39]. When overweight and obese patients were examined, while LV-EF (and RV-EF) were similar by weight categories, global LV-CS differed significantly [40]. LV asynchrony may exist in rTOF patients with right bundle branch block, which is associated with a reduced regional and global LV function [41]. In the presence of normal LV-EF, LV-CS was found to be decreased at the LV base and apex suggesting intraventricular dyssynchrony [42]. Moreover, higher LV (and RV) wall motion delay as dyssynchrony parameters were associated with lower peak oxygen consumption and worse LV-EF and RV-EF values [43].

The residual pulmonary regurgitation (PR) following TOF repair mediates biventricular dysfunction/dyssynchrony affecting long-term adverse outcomes [44]. A fall-and-rise pattern for global LV-LS and RV-LS could be detected following TOF repair, which was not seen in the case of patients undergoing PVR [45]. In another study, global LV-LS and RV-LS improved significantly 6 months after PVR [46]. Global, basal and apical LV-CS and basal synchrony showed improvement with no change in RV global strains following PVR [47]. The LV strain and strain rate before PVR have important prognostic power in predicting adverse events after PVR in the presence of rTOF [48].

Global LV-LS (and RV-LS) were associated with adverse cardiac events in rTOF [49]. In a cMRI-derived feature tracing (FT) study, LV-RS, LV-CS and LV-LS (and RV-LS) were related to mortality [50]. In another cMRI-FT study, LV-CS rate was an independent predictor of sustained/non-sustained ventricular tachycardia requiring invasive treatment [51].

##### LV Rotational Mechanics

Abnormalities of the LV rotational mechanics are known characteristics in rTOF, but the results are unclear [10,27,52]. In the MAGYAR-Path Study, 38% of rTOF patients showed absence of normally directed LV rotational mechanics called LV ‘rigid body rotation’, from which 27% were clockwise oriented and 11% were counterclockwise oriented [10]. These results in other studies proved to be 15% and 18% [27]. In another study, 38% of rTOF patients had reversed [counterclockwise] LV basal rotation [52].

We found that 62% of rTOF patients had LV rotational mechanics in a normal direction; in these cases, impaired LV apical rotation was associated with preserved LV basal rotation [10]. In another study, only 10% of rTOF patients showed reduced LV apical rotation [27]. Moreover, LV apical rotation was reduced, but not reversed in another paper [52]. An association of decreased LV apical rotation with worse outcomes in rTOF patients [49]. According to the findings from the MAGYAR-Path Study, increased aortic stiffness was associated with reduced LV apical rotation [10].

### 3.2. Left Atrium

#### 3.2.1. Under Healthy Circumstances

**Figure 2:**
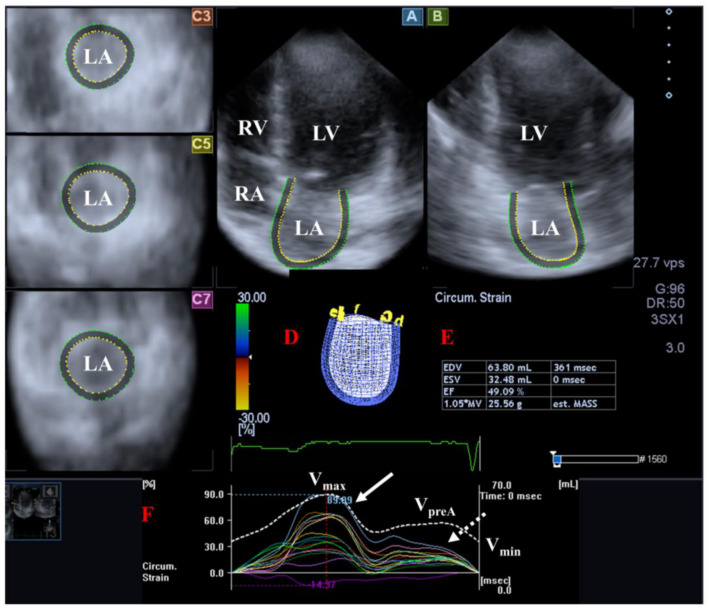
Examination of the left atrium [LA] by three-dimensional [3D] speckle-tracking echocardiography. Following echocardiographic data acquisitions, the following typical views are created: (**A**) Apical 4-chamber and (**B**) two-chamber longitudinal views and (**C3**,**C5**,**C7**) cross-sectional views at basal, midatrial and superior levels, respectively. LA can be easily detected alongside other heart cavities including the left ventricle [LV] and the right atrium [RA] and ventricle [RV]. A number of other details were also presented including (**D**) 3D cast, (**E**,**F**) maximum [V_max_], preatrial contraction [V_preA_] and minimum [V_min_] volumes of the LA and curves representing changes in volumes and strains of LA over time. Reservoir [peak] and active contraction LA strains are represented by white and dashed white arrows, respectively.

The muscle fibers of the LA run in circumferential and longitudinal directions. There are several phases of LA function including reservoir (in systole, its volume is highest), conduit (in early diastole) and booster pump (in late diastole, its volume is lowest). The Frank–Starling mechanism has significance in shaping the function of LA [53,54,55] (Figure 2).

#### 3.2.2. In Repaired Tetralogy of Fallot

Abnormal LA deformation was found in rTOF patients [11,23,56,57,58]. In recent findings from the MAGYAR-Path Study, increased LA volumes respecting the cardiac cycle could be demonstrated, which were accompanied by reduced LA total, passive and active emptying fractions and preserved LA stroke volumes. From peak LA strains representing the LA reservoir phase, global and mean segmental LA-CS, LA-LS and LA-AS were decreased. From LA strains at atrial contraction, all global LA strains (RS, CS, LS, AS and 3DS) were found to be reduced. These findings draw attention to the fact that all phases of LA function are compromised in adult TOF patients late after repair [11]. This was partly confirmed in a later study, in which abnormal reservoir LA strain and LA compliance could be demonstrated in adult rTOF patients [56]. rTOF patients had reduced peak LA-LS, LA contraction strain and LA ejection fraction. Moreover, peak LA-LS correlated negatively with RA end-diastolic volume normalized to body surface area, whereas LA-EF correlated weakly with LV-EF as well [23].

### 3.3. Mitral Valve

#### 3.3.1. Under Healthy Circumstances

**Figure 3:**
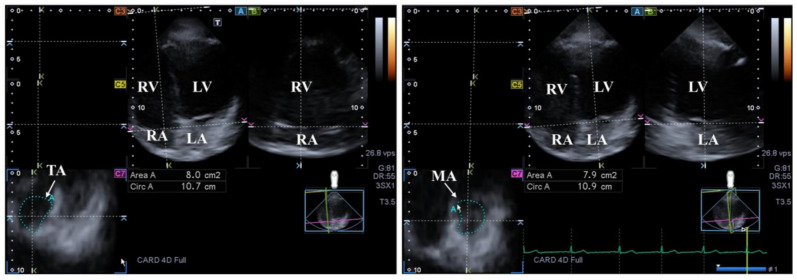
Examination of the tricuspid (TA) and mitral (MA) annuli by three-dimensional (3D) echocardiography (left panel and right panel, respectively). (**A**) Apical 4-chamber and (**B**) two-chamber longitudinal views help visualization of valvular annuli on (**C7**) cross-sectional view. TA and MA planes are marked by a white arrow. TA and MA can be easily detected alongside the heart chambers including the left ventricle (LV) and atrium (LA) and the right ventricle (RV) and atrium (RA).

The MV has several components: two leaflets, a subvalvular apparatus consisting of chordae tendinae and papillary muscles and a saddle-shaped annulus (MA) that has a dynamic motion respecting the heart cycle. The MV opens/closes during diastole/systole with one-way flow of blood from the LA into the LV in normal healthy circumstances. Adjacent regions of these heart chambers have a significant role in the contraction of MV [16,59,60] (Figure 3).

#### 3.3.2. In Repaired Tetralogy of Fallot

In a recent study from the MAGYAR-Path Study, dilation and dysfunctional MA could be detected in adult rTOF patients. It could be stated that TOF patients who underwent early palliation/later correction had worse results as compared to cases with early total reconstruction. The age at the time of early total reconstruction and MA systolic dimensions correlated as well. The ratio of grade 1–2 mitral regurgitation was 24%, and no subjects had a higher grade of mitral regurgitation [13].

### 3.4. Aortic Valve

#### 3.4.1. Under Healthy Circumstances

The AV consists of three semilunar leaflets; it opens/closes during ventricular systole/diastole. In healthy circumstances, there is a one-way LV-aortic flow [20].

#### 3.4.2. In Repaired Tetralogy of Fallot

An association could be demonstrated between increased ascending aortic dimension and aortic valve regurgitation in rTOF patients [61].

### 3.5. Aorta

#### 3.5.1. Under Healthy Circumstances

The aorta is the central element of the systemic circuit, the largest artery with characteristic distensibility/stiffness features. The aorta and LV interact closely with each other [coupling] [62,63].

#### 3.5.2. In Repaired Tetralogy of Fallot

Possibly, abnormal histopathology of the aortic media may be behind the aortic dilation in TOF, which can lead to regurgitation, dissection, or rupture due to its role in weakening the aortic wall [64,65]. TOF patients show abnormal aortic features correlating with higher age, which may be associated with later repair [66]. Interestingly, abnormal aortic elastic property is found to be confined to the proximal [not distal] segments regardless of the ope rative status [67]. Children with postoperative TOF have stiffer aortas [68]. Despite early repair and normal aortic dimensions, preadolescents and adolescents with TOF had elevated wall shear stress, increased stiffness, and pathologic systolic flow formations in the proximal aorta, suggesting that although early repair normalizes aortic dimensions in childhood, TOF patients remain at risk for late aortic complications [69]. High prevalence of aortic dilation and stiffness as assessed by pulse wave assessment, echocardiography and cMRI are found in rTOF patients [70,71,72,73,74]. Male sex influences and is the strongest factor for aortic dilation [70,71]. In a recent stress cMRI study, reduced aortic distensibility during exercise could be detected in rTOF [26]. Patients with rTOF have lower ascending aortic distensibility, higher aortic stiffness index and lower global peak circumferential ascending aortic strain assessed by 2D-STE compared to controls [75]. As mentioned above, aortic strains correlated with LV strains [9] and LV apical rotation [10]. Interestingly, increased aortic stiffness was associated with decreased LV apical rotation in rTOF. This result is contrary to what can be found in healthy subjects, where increased aortic stiffness is positively correlated with LV apical rotation, suggesting an abnormal physiologic response in rTOF [10]. Dilation and stiffening of the ascending aorta were frequent findings in repaired patients with complex CHD including TOF, which was associated with diminished exercise capacity and morbidity [76]. If the balance between the blood supply and the workload of the heart is examined, its maintenance can be confirmed regardless of the stiffness of the aorta in rTOF [77]. Matrix metalloproteinases, which are capable of degrading extracellular matrix proteins, polymorphism of MMP-9 (not MMP-3) has an influence on aortic stiffness and root dilation [78]. In CHD patients, including those with rTOF, increased transforming growth factor-beta 1 (TGF-ß1) levels were present, which correlated with aortic sinus dimension [79]. Moreover, autonomic cardiac function is impaired in rTOF patients, which is independently associated with vascular function represented by carotid artery stiffness [80].

Early studies indicated abnormal arterial haemodynamics after TOF repair [81]. According to recent findings, abnormal aortic flow seen in rTOF is associated with increased viscous energy loss in the thoracic aorta, the magnitude of which is associated with LV function and volumes. It is theorized to be due to inherently abnormal LV outflow geometry and/or RV interaction. Reduced aortic flow efficiency increases cardiac work and may be an important factor in long-term cardiac performance [82].

## 4. The Right Heart and the Pulmonary Artery

### 4.1. Right Ventricle

#### 4.1.1. Under Healthy Circumstances

The RV is not similar to the LV; its shape, when viewed from the front, resembles a triangle while its cross-sectional image resembles a crescent moon. The RV encircles the LV, its wall thinner than the LV (only 3–5 mm), and trabecularizations are more pronounced in the RV apex than in the LV apex [83]. Contraction of the RV starts in the inlet and ends in the outflow tract. The free wall of the RV contains superficial subepicardial fibers arranged in the transverse direction and deep subendocardial fibers, which run longitudinally and extend from the base toward the apex. While longitudinal shortening is due to longitudinal fibers, RV free-wall radial movement is due to fibers running in a circumferential direction. There is a ventricular interdependence of LV and RV due to superficial fibers as well. Heart rate, Frank–Starling mechanism and autonomic nervous system have significant roles in the determination of RV function, which has no rotational/twisting components [83,84,85,86].

#### 4.1.2. In Repaired Tetralogy of Fallot

In rTOF, the dimensions and function of the RV have major concerns [30]. It has been confirmed that both systolic and diastolic RV function deteriorated shortly after surgery [39,87]. Global RV function and exercise capacity were similarly impaired regardless of the presence of rTOF in patients with a severely dilated RV [88]. The RV myocardial systolic-to-diastolic duration ratio incorporates systolic and diastolic performance, electromechanical dyssynchrony, and postsystolic shortening and is associated with exercise capacity in rTOF [89]. Although Doppler parameters proved to be normal, adults following late after TOF repair still showed deteriorated RV myocardial function as assessed by tissue Doppler imaging [90]. In pediatric rTOF patients, global RV-LS was decreased, while RV transverse strain was increased in patients with normal EF [91]. Patients with rTOF had LV, RV and interventricular dyssynchrony, which showed no correlations with changes in ventricular size and function over time [92]. RV longitudinal pumping was associated with LV filling pressure in rTOF patients explaining LV underfilling in patients with PR [93]. Late after repair in adults, reduced RV free-wall strain and strain rate were present, especially at the apical region, suggesting that this is the most affected RV region [35]. Interestingly, surrogates of RV dyssynchrony did not show correlations with outcomes in adults with rTOF [94]. In another study, reduced RV (and LV) early diastolic strain rate could also be detected in rTOF [37]. The diameter of the RV outflow tract (RVOT) increased gradually at all ages, but in the first decade after surgery, this turned out to be more pronounced. More rapid RVOT enlargement was noted in patients with a larger RV, more PR, and greater rates of increases in RV size and PR severity [95].

Regarding prognostication, RV (and LV) deformation is of prognostic significance and has significantly improved risk stratification in terms of RV size and certain variables [96]. Both global RV-LS and RV free-wall LS were found to be associated with adverse events [49,97], RV free-wall LS provided superior prognostic value than that of global RV-LS in rTOF patients [97]. These findings were strengthened by others confirming that TAPSE and RV strain worsen following TOF repair in children together with LV parameters, possibly having prognostic implications [34].

It has been confirmed that in patients with PR and residual RV outflow tract obstruction had smaller RV volumes and higher RV-EF [98]. Others found that residual RV outflow tract obstruction does not affect RV function [99] or have increased RV-CS and RV-RS [100]. Previous PVR showed no association with changes in RV-EF, but with an increased risk of infective endocarditis and atrial arrhythmias [101].

### 4.2. Right Atrium

#### 4.2.1. Under Healthy Circumstances

**Figure 4:**
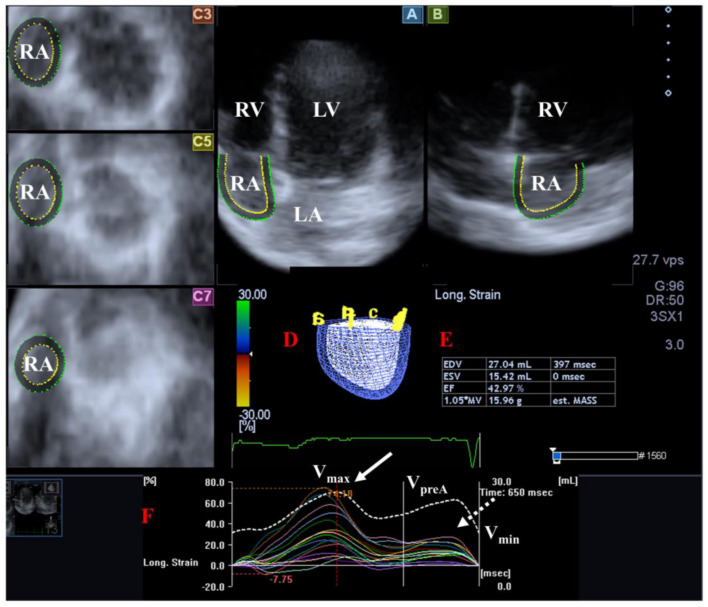
Examination of the right atrium (RA) by three-dimensional (3D) speckle-tracking echocardiography. Following echocardiographic data acquisitions, the following typical views are created: (**A**) Apical 4-chamber and (**B**) two-chamber longitudinal views and (**C3**,**C5**,**C7**) cross-sectional views at basal, midatrial and superior levels, respectively. RA can be easily detected alongside other heart cavities including the left ventricle (LV) and atrium (LA) and the right ventricle (RV). A number of other details were also presented including (**D**) 3D cast and (**E**,**F**) maximum (V_max_), preatrial contraction (V_preA_) and minimum (V_min_) volumes of the RA and curves representing changes in volumes and strains of _RA over time. Reservoir (peak) and active contraction RA strains are represented by white and dashed white arrows, respectively.

The RA is composed of three components: the venous part, the appendage and the vestibule. The muscle fibers of the RA run in circumferential and longitudinal directions. There are several phases of RA function including reservoir [in systole, its volume is highest], conduit [in early diastole] and booster pump [in late diastole, its volume is lowest]. Additionally, RA has a regulator role in the conduction of the heart via the sinus node which is located in its wall and also produces atrial natriuretic peptides regulated by tension and baroreceptors [102] (Figure 4).

#### 4.2.2. In Repaired Tetralogy of Fallot

RA end-diastolic volume, RA-EF and RA-LS representing reservoir function are abnormal in TOF. These abnormalities may indicate the presence of an RA diastolic burden due to chronic RV dysfunction in the presence of rTOF [103]. Increased RA volume was observed in adult rTOF patients whose higher RA volumes were associated with a higher incidence of supraventricular arrhythmia, which was more frequent in men and in patients with reduced LV-EF [104]. rTOF patients had reduced peak RA-LS, RA contraction strain and RA-EF, moreover, peak RA-LS and mean RV strain were associated [23]. In ventricular systole, early diastole and atrial contraction lower RA (and LA) peak positive and total strain could be detected suggesting impaired atrial mechanics in rTOF [57]. Results from the MAGYAR-Path Study confirmed the complexity of RA dysfunction [12]. All RA volumes respecting the cardiac cycle were increased, while total and passive RA emptying fractions were reduced with preserved active RA emptying fraction and all RA-SVs. From peak reservoir RA strain, global RA-RS, RA-LS and RA-AS were reduced, while from RA strains at atrial contraction, only global RA-CS and RA-3DS were decreased [12]. While adverse events could be independently predicted by RA dilation [105], RA (and RV) strain was an independent predictor of arrhythmic events among patients with rTOF [106].

### 4.3. Tricuspid Valve

#### 4.3.1. Under Healthy Circumstances

The tricuspid valve has several components: three leaflets, a subvalvular apparatus consisting of chordae tendinae and papillary muscles and an asymmetrical, saddle-shaped, ellipsoid annulus (TA) that has a dynamic motion respecting the heart cycle. The tricuspid valve opens/closes during diastole/systole with a one-way flow of blood from the RA into the RV in normal healthy circumstances [107] (Figure 3).

#### 4.3.2. In Repaired Tetralogy of Fallot

The diameter of the tricuspid ring was found to be increased in rTOF [90]. In accordance with these findings, dilated TA with reduced functional properties could be demonstrated in adult patients with rTOF in the MAGYAR-Path Study. Moreover, TA dilation was related to RA volumes. Interestingly, when the results of TOF patients with early total reconstruction and early palliation/late correction were compared, similar TA dimensions and TA functional properties could be demonstrated [14]. In this study, 83% of rTOF patients had grade 1–2 tricuspid regurgitation (TR) with a minimal number of patients having higher grade TR, predominantly in cases with early palliation/late correction [14].

### 4.4. Pulmonary Valve

#### 4.4.1. Under Healthy Circumstances

Similarly to AV, the pulmonary valve (PV) is morphologically semilunar and has three leaflets and separates the RV from the pulmonary artery. The PV opens/closes at ventricular systole/diastole to control one-way blood flow [108].

#### 4.4.2. In Repaired Tetralogy of Fallot

In the presence of PR, RV dilation, dysfunction and/or dyssynchrony may gradually develop during long-term follow-up leading to RV failure [44]. In rTOF, PR and resulting RV and LV dysfunction are associated with adverse clinical outcomes [46]. In rTOF patients with pulmonary stenosis >50% earlier PVR would be beneficial, which does not depend solely on RV size and EF, global RV-LS seems to be a more sensitive marker [109].

### 4.5. Pulmonary Artery

#### 4.5.1. Under Healthy Circumstances

The primary role of the pulmonary artery (PA) is to carry deoxygenated blood from the RV to the pulmonary arterial system. The pulmonary artery is a low-pressure low-resistance system. Similarly to the left heart, there is a significant interaction between RV contractility and RV afterload called RV–PA coupling [86].

#### 4.5.2. In Repaired Tetralogy of Fallot

Similarly to the aorta, vascular dysfunction of the PA could be detected in rTOF represented by elevated PA elastance. It showed associations with exercise intolerance and an inverse correlation with the severity of PR, which may prevent PR and RV and LV dilation when significant pulmonary stenosis does not exist [110]. Impaired RV–PA coupling was found in rTOF patients, which was mainly affected by the strategy used at the primary surgery [111].

## 5. Pathophysiological Background

In short, the majority of the abnormalities detailed above can basically be traced back to the basics of the disease, heart failure is most commonly caused by pulmonary regurgitation, pulmonary stenosis, dilation of the RV, LV dysfunction or aortic regurgitation. In addition, conditions that develop during surgical procedures can also have an effect. In case of early palliation/late correction, the consequences of the systemic-pulmonary shunt and those of the consequential LV volume overload are seen, while patients with an early total reconstruction may require intervention or reoperation of the pulmonary homograft. The mutual effects of the heart chambers, valves and the great vessels must not be forgotten either. In addition to these, of course, not only the surgical procedure itself and its timing may also play a role [1,2,3,44].

## 6. Novelty of the Present Review

To the best of the author’s knowledge, this is the first review that tried to summarize the most important findings related to myocardial, valvular and vascular abnormalities in patients following rTOF. Although previous articles and reviews have attempted to collect available scientific data on this topic, they have generally examined the topic according to a specific aspect (e.g., the method used). In addition, valvular and vascular abnormalities were not investigated in conjunction with myocardial abnormalities, as this review aimed to do.

## 7. Clinical Implications

Although TOF is a rare disorder, there are special clinical consequences related to this pathology following its repair. Knowing these potential complications helps in the early diagnosis and leads to the creation of protocols used in the management of patients in the determination of the ideal time of reoperation. There have been significant improvements in surgical strategies, perioperative care and cardiovascular imaging over the past decades, which enabled these patients to live longer and have a better quality of life.

## 8. Conclusions

The cardiac chambers of patients with rTOF show significant volumetric and functional (strain, rotational, etc.) abnormalities, which are associated with significant valvular and vascular abnormalities as well (Table 1). There are pieces of evidence from clinical studies showing that early total reconstruction is associated with beneficial results during a long-term follow-up. These findings partially suggest their importance in the improvement of late complications in rTOF. Therefore, further evidences are required to compare different surgical strategies on late morbidity data. Moreover, advanced imaging techniques could help to detect specific subclinical abnormalities, whose clinical and prognostic importance should be clarified in rTOF as well.

## Figures and Tables

**Table 1:** Summarization of the most important findings.

		References
LEFT HEART		
Left ventricle	There is a strong interaction between LV and RV function	[23,24]
	rTOF is associated with impaired LV deformation represented by LV strains	[9] *, [27,28,29,30,31,32,33,34,35,36,37,38,39,40,41,42,43,44,45,46,47,48,49,50,51]
	There are abnormalities of the LV rotational mechanics in rTOF	[10] *, [27,49,52]
Left atrium	LA volumes are increased with reduced LA-EFs and preserved LA-SVs	[11] *
	LA strains are reduced in rTOF	[11] *, [23,56]
Mitral valve	MA is dilated and dysfunctional in rTOF with mild and low ratio of mitral regurgitation	[13] *
Aortic valve	Aortic valve regurgitation and increased ascending aortic dimension are associated in rTOF patients	[61]
Aorta	rTOF patients show abnormal aortic features including dilation and increased stiffness	[64,65,66,67,68,69,70,71,72,73,74,75,76,77,78,79,80]
RIGHT HEART		
Right ventricle	Both systolic and diastolic RV function are deteriorated in rTOF	[30,34,35,37,49,87,88,89,90,91,92,93,94,95,96,97,98,99,100,101]
Right atrium	RA volumes and strains are abnormal in rTOF	[12] *, [23,57,103,104,105,106]
	All RA volumes respecting the cardiac cycle were increased, while total and passive RA emptying fractions were reduced with preserved active RA emptying fraction and all RA-SVs.	[12] *
Tricuspid valve	Dilated TA with reduced functional properties are present in rTOF mostly only with grade 1–2 TR	[14] *
Pulmonary valve	In the presence of PR, RV dilation, dysfunction and/or dyssynchrony may gradually develop	[44]
	In rTOF patients with PS, earlier PVR would be beneficial	[109]
Pulmonary artery	Vascular dysfunction of the PA could be detected in rTOF	[110,111]

Abbreviations. LA = left atrium, LV = left ventricle, PR = pulmonary regurgitation, PS = pulmonary stenosis, PVR = pulmonary valve replacement, RA = right atrium, rTOF = repaired tetralogy of Fallot, RV = right ventricle, SV = stroke volume, TR = tricuspid regurgitation. The star [*] represents studies from the MAGYAR-Path Study. In some topics, results are contradictory.

## Data Availability

Not applicable.
